# Comparative study of acute-phase traumatic brain injury in rats of different weeks of age under identical impact force

**DOI:** 10.3389/fneur.2026.1928195

**Published:** 2026-10-19

**Authors:** Diyou Chen, Peng Wang, Letian Zhang, Jinlong Qiu, Xiyan Zhu, Hongyi Xiang, Qiushi Wang, Yu Guo, Hui Zhao, Kunlin Xiong

**Affiliations:** 1 Department of Radiology, Daping Hospital, Army Medical University, Chongqing, China; 2 Chongqing Clinical Research Centre of Imaging and Nuclear Medicine, Chongqing, China; 3 Chongqing Municipal Key Clinical Specialty (Excellence) Construction Platform, Chongqing, China; 4 State Key Laboratory of Automotive Simulation and Control, Jilin University, Changchun, Jilin, China; 5 Institute for Traffic Medicine, Daping Hospital, Army Medical University, Chongqing, China

**Keywords:** behavioral changes, biomechanical responses, closed head injury, rat age, traumatic brain injury

## Abstract

The closed head injury (CHI) model in rats is widely used in preclinical research, yet this model exhibits certain variability, partly due to the use of rats of different ages in experiments. To explore this variability, we selected rats of commonly used ages (8, 10, and 12 weeks) and induced brain injury under the same impact strength, comparing biomechanical responses, behavioral changes, and neuropathological outcomes. The results indicated that with increasing age, linear acceleration and rotational acceleration significantly decreased, and the younger age group (8 weeks) exhibited more severe neurological deficits and behavioral impairments, particularly in motor coordination and anxiety-like behaviors. Histological analysis showed the most severe neuronal loss and disorganization in the cerebral cortex and hippocampus in the 8-week group, with more extensive axonal injury. In contrast, the 10-week and 12-week groups showed milder pathological changes and better-preserved neuronal morphology. Molecular analysis revealed higher expression levels of glial fibrillary acidic protein, cleaved caspase-3, and interleukin-1 beta in younger rats, suggesting a positive correlation between these molecular markers and injury severity. Overall, this study reveals the significant impact of age differences in rats on TBI outcomes, highlighting the importance of age in TBI experiments and providing a basis for reducing experimental variability and enhancing the translational relevance to clinical applications.

## 1 Introduction

Traumatic brain injury (TBI) is a major global public health concern, affecting more than 69 million people worldwide each year (1, 2). TBI frequently results in a range of long-term sequelae, including motor dysfunction, cognitive decline, emotional disturbances, and impaired social functioning, all of which significantly compromise patients’ quality of life and social integration (3–5). Moreover, it imposes substantial caregiving burdens and emotional stress on families (6). Given the significant individual variability in humans and the complex and dynamic pathological pro cesses of TBI, clinical studies face numerous ethical and methodological limitations. Therefore, establishing scientifically valid and controllable animal models is crucial for elucidating the underlying mechanisms of TBI.

Among the currently available animal models for TBI, rats have become one of the most widely used experimental subjects due to their low cost, ease of maintenance, and physiological and pathological responses that closely resemble those of humans (7, 8). Existing TBI models are generally classified into two main categories: open head injury (OHI) and closed head injury (CHI) models (7, 9–11). Compared to OHI models, CHI models do not require craniotomy; instead, they apply controlled mechanical force directly to the intact skull, inducing instantaneous acceleration or deceleration of the head. This leads to shear stress and axonal stretching in brain tissues, thereby simulating real-world brain injury scenarios such as falls or motor vehicle accidents with greater clinical relevance (11). As a result, CHI models are widely adopted for studies investigating the pathophysiology of TBI and evaluating potential therapeutic interventions.

Although the CHI model has been widely applied in TBI research, considerable variability persists across studies in both histopathological assessments and neurological outcomes. Differences in animal age, together with the associated variations in body weight, may represent one potential source of this variability. Previous studies have reported broad body weight ranges within the same experimental paradigms, for example 250–350 g, 250–400 g, and 340–540 g (12–16), which generally correspond to rats aged approximately 8–12 weeks. Evidence indicates that animals at different developmental stages may exhibit distinct injury characteristics. For example, Zvejniece et al. (17), using a weight-drop model, observed that older mice possessed thicker parietal bones and a reduced susceptibility to skull fracture. Similarly, Guilhaume-Correa et al. (18) compared TBI outcomes between 6-week-old and 9-week-old mice and found higher mortality and more pronounced hippocampal neuronal damage in the younger cohort. Collectively, these findings suggest that age may influence head injury outcomes and should be considered in experimental TBI studies.

To systematically investigate the effect of weeks of age on TBI outcomes in rats, this study employed a CHI model to compare injury profiles across three commonly used age groups (8, 10, and 12 weeks of age, corresponding to approximately 200 g, 300 g, and 400 g, respectively) under standardized conditions. TBI was induced by a pneumatically driven impactor, and real-time biomechanical responses of the head—including linear acceleration and angular velocity—were recorded via embedded sensors. Comprehensive post-injury assessments were performed to evaluate neurological function, behavioral outcomes, and neuropathological changes. This study provides new insights into how weeks of age influences TBI outcomes in preclinical models, offering a basis for better understanding variability in injury responses and for improving the consistency and translational value of experimental brain injury research.

## 2 Methods and materials

### 2.1 Ethical review

The animal experimental design, procedures, and euthanasia methods were approved by the Institutional Animal Care and Use Committee of Army Medical University (Approval No. AMUWEC20210836) and were conducted in accordance with ethical guidelines for animal welfare.

### 2.2 Animals prepare

Male Sprague–Dawley rats aged 8–12 weeks and weighing approximately 180–410 g were obtained from the Animal Experiment Center of Daping Hospital. Prior to the experiments, all animals were acclimatized for one week under standard laboratory conditions, including a temperature of 22 ± 2 °C, relative humidity of 55–65%, and a 12-h light/dark cycle, with food and water available ad libitum. The experimental cohort consisted of 48 rats. Based on age, the rats were divided into 8-week (approximately 180–220 g), 10-week (approximately 280–310 g), and 12-week (approximately 370–410 g) cohorts, as shown in Figure 1A. Each age cohort was further divided into sham and TBI groups, with eight rats in each group (*n* = 8), resulting in six experimental groups. All eight rats in each group underwent behavioral assessments. After behavioral testing, five rats from each group were randomly selected for histopathological examination, and the remaining three rats were used for Western blot analysis.

### 2.3 TBI procedure

Rats were subjected to a cranial impact on the vertex using a pneumatically driven BIM-IV impactor, as previously described (19). Anesthesia was induced with isoflurane (4.5%) and maintained with 2.5–3% in oxygen (0.9 L/min). After shaving the scalp, a steel helmet (10 mm in diameter and 2 mm thick) was affixed to the midline between bregma and lambda to prevent skull fracture. Angular velocity and linear acceleration sensors were mounted in a mold, which was bonded with cyanoacrylate adhesive to the anterior part of the helmet at approximately 5 mm distance, and further secured with adhesive tape. The cylinder piston is pulled to align the impact rod with the helmet, with a distance of 5 cm between them. A driving pressure of 0.7 MPa was selected to generate a relatively severe injury condition with sufficiently robust acute behavioral and neuropathological changes for intergroup comparisons (19, 20). The data acquisition system of the sensors was manually triggered 3 s prior to impact. Immediately after impact, isoflurane administration was discontinued, and the animals were transferred to a preheated warming pad maintained at 37 °C to preserve body temperature during recovery. Respiratory status was closely monitored. Mechanical ventilation was provided only to rats exhibiting apnea or marked respiratory depression and was continued until stable spontaneous respiration resumed. No additional analgesic medication was administered after injury, and no pain-specific assessment was performed. Rats were returned to their home cages after stable spontaneous respiration and spontaneous activity had recovered.

Head kinematic data during impact were recorded by a Donghua sensor system at a sampling rate of 10 kHz. To remove noise, the raw data were filtered using a Channel Frequency Class 1,000 (21). Rotational acceleration was obtained as the first derivative of angular velocity with respect to time.

### 2.4 Behavioral assessments

Behavioral assessments were conducted at predefined time points. On the day of injury, the loss of righting reflex (LRR) was recorded immediately after impact, and the modified Neurological Severity Score (mNSS) was assessed at 1 h post-TBI. On the following day, the rotarod test was conducted at 24 h post-TBI, followed by the elevated plus maze (EPM) test after a 30-min recovery interval. The same testing sequence and time intervals were applied to all experimental groups.

Immediately after injury, rats were subjected to the LRR test. LRR was defined as the time interval from isoflurane discontinuation to the first sign of righting after impact (22). At 1 h post-TBI, neurological impairment was evaluated using the mNSS (20). The mNSS consists of ten different tasks assessing motor function, balance, and alertness. Failure to perform a task was scored as 1 point, whereas successful performance received 0 points. Thus, the maximum score of 10 indicates severe neurological deficits, with failure in all tasks.

Motor coordination was evaluated 24 h post-injury using a rotarod apparatus with a diameter of 90 mm. After a 2-min acclimatization period at constant speed (4 rpm), rats underwent four 5-min trials with 10-min inter-trial intervals (11). During testing, the rod accelerated from 4 to 40 rpm over 5 min. Latency to fall was recorded as the primary metric of motor coordination.

After completion of the rotarod test and a 30-min recovery interval, anxiety-like behavior was assessed using the EPM test (23). The apparatus consisted of two open arms (50 × 10 cm) and two closed arms (50 × 10 × 40 cm) arranged in a cross-shaped configuration and elevated 60 cm above the floor, as shown in Figure 1B. Each rat was placed in the central area (10 × 10 cm) facing an open arm and allowed to freely explore for 5 min. After each trial, the apparatus was cleaned with 75% ethanol to eliminate olfactory cues. The number of entries into each arm and the time spent in each arm were recorded using Tracking Master software. A reduction in the number of open-arm entries and the duration spent in the open arms was considered indicative of anxiety-like behavior.

**Figure 1:**
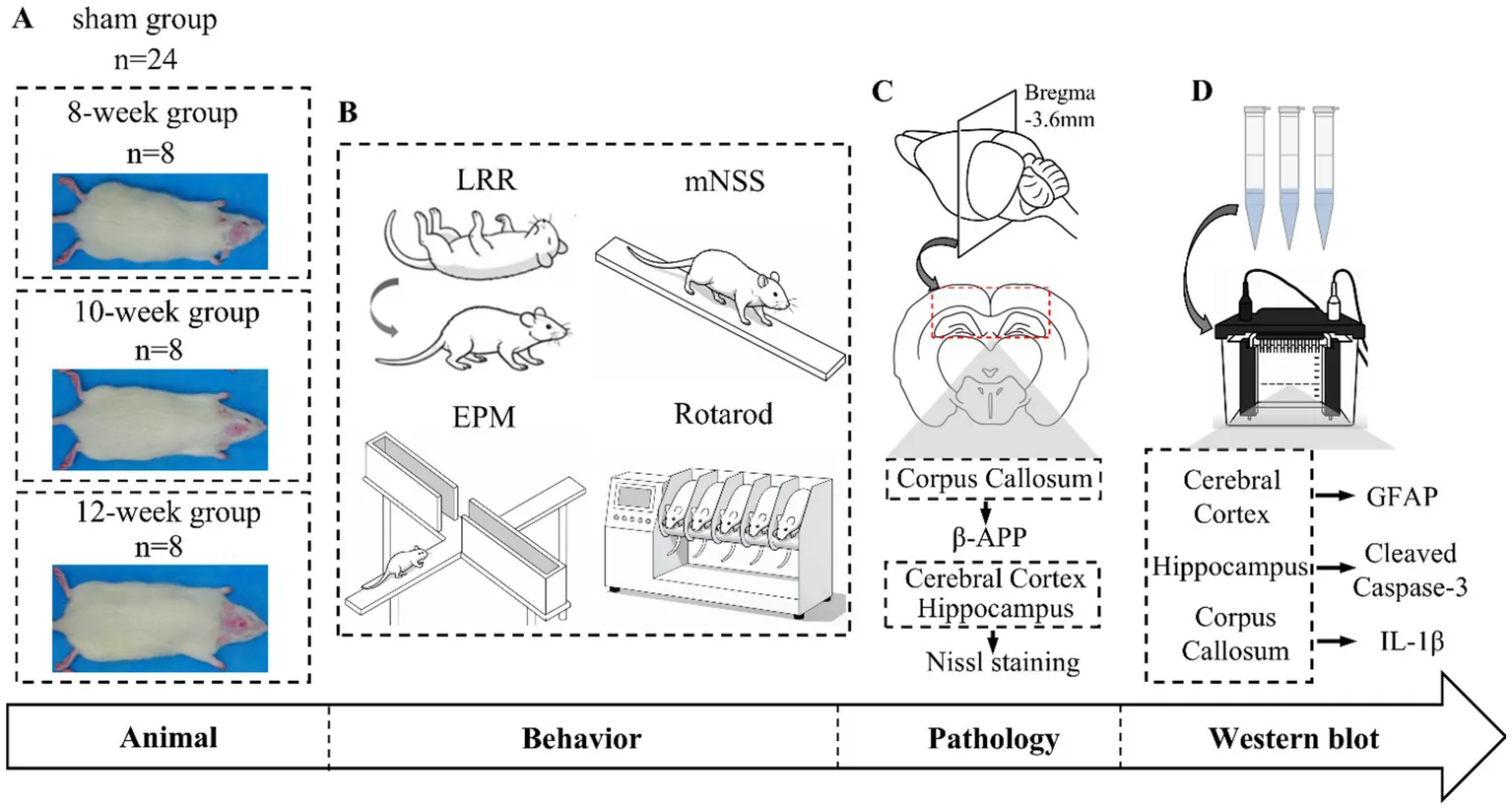
Overview of methodology. **(A)** Animal prepare: grouping and sample size of rats. Following head impact, the rats were sequentially subjected to the LRR, mNSS, rotarod, and EPM tests, as illustrated in **(B)**. **(C)** After the behavioral tests, five rats were randomly selected for pathological examination. Tissue blocks were taken at Bregma −3.6 mm, embedded in paraffin, and sectioned for Nissl and β-APP staining. **(D)** The remaining rats underwent Western blot analysis. The cortex, hippocampus and corpus callosum were dissected, followed by quantitative protein assays for GFAP, cleaved caspase-3, and IL-β.

### 2.5 Histopathology

At the experimental endpoint, following completion of behavioral testing, rats assigned to histopathological examination were euthanized by an intraperitoneal overdose of sodium pentobarbital (120 mg/kg). The animals were subsequently transcardially perfused, and the brains were collected as previously described (19). The harvested brains were fixed in 4% paraformaldehyde solution for at least 12 h, dehydrated, embedded in paraffin, and serially sectioned at a thickness of 4 μm.

Histopathological analysis was performed in sham animals (*n* = 5) and injured animals (*n* = 5), with at least three sections examined per animal. To evaluate neuronal damage in the cortical and hippocampal regions, sections were subjected to Nissl staining. After deparaffinization, sections were stained with Cresyl violet for 10 min, followed by graded alcohol dehydration, xylene clearing, and coverslipping, as shown in Figure 1C. Stained sections were observed under a light microscope, and neuronal counts were conducted at 40 × magnification to assess cell loss.

Axonal injury in the corpus callosum was assessed using beta-amyloid precursor protein (β-APP) immunohistochemistry. Following deparaffinization and rehydration, sections were incubated in 3% H₂O₂ to block endogenous peroxidase activity and subjected to citrate antigen retrieval (95 °C, 15 min). After blocking, sections were incubated overnight at 4 °C with primary antibody against β-APP (1:500 dilution). On the following day, HRP-conjugated secondary antibody was applied, and visualization was achieved with DAB chromogen, followed by hematoxylin counterstaining and coverslipping. Images were captured under a light microscope, and positive signal distribution and density were analyzed.

### 2.6 Western blot

At the experimental endpoint, rats assigned to Western blot analysis (*n* = 3 per group) were euthanized by an intraperitoneal overdose of sodium pentobarbital (120 mg/kg). The cerebral cortex, corpus callosum, and hippocampus were then rapidly dissected, snap-frozen in liquid nitrogen, and stored at −80 °C. Tissue samples were lysed in RIPA buffer, and total protein was extracted. Following quantification of protein concentration, 40 μg of protein per sample was subjected to SDS-PAGE, and the separated proteins were transferred onto polyvinylidene fluoride membranes, as shown in Figure 1D. Membranes were incubated overnight at 4 °C with the following primary antibodies: glial fibrillary acidic protein (GFAP; 1:1,000), cleaved caspase-3 (1:1,000), and interleukin-1 beta (IL-1β; 1:1,000). The following day, the membranes were incubated with horseradish peroxidase-conjugated secondary antibodies for 1 h at room temperature. Protein bands were visualized using enhanced chemiluminescence and acquired with a chemiluminescence imaging system. β-actin was used as the loading control, and band intensities were quantified using ImageJ.

### 2.7 Statistical analysis

Statistical analyses were performed using Origin 2018. Data are presented as the mean ± SD, except for head kinematic time-history curves (mean with 95% confidence intervals) and mNSS data (median with interquartile range). The mNSS data were analyzed using the Kruskal–Wallis test followed by Dunn’s *post hoc* test. Other behavioral, histopathological, and Western blot outcomes were compared among the six groups using one-way ANOVA followed by Tukey’s *post hoc* test. Head kinematic parameters, head mass, and skull thickness were similarly compared among the three injured age groups. Statistical significance was set at *p* < 0.05. Asterisks indicate comparisons with the corresponding age-matched sham group (*p < 0.05, **p < 0.01, and ***p < 0.001), whereas hash symbols indicate comparisons among injured groups (#*p* < 0.05, ##*p* < 0.01, and ###*p* < 0.001).

## 3 Result

### 3.1 Mortality rate

A total of 55 rats were used in the experiment, with 7 rats dying, resulting in a mortality rate of 12.7%. Under the same impact strength (0.7 MPa), there were three deaths in the 8-week group, two in the 10-week group, and two in the 12-week group. Despite prompt resuscitation attempts, these animals ultimately died due to prolonged apnea. To maintain the predetermined group sizes, additional rats were subsequently included to replace the animals that died.

### 3.2 Biomechanical responses, head mass, and skull thickness

To compare age-related differences in head kinematics among rats under identical impact strength, motion sensors were used to record the translational and rotational loading experienced by the head, as shown in Figures 2A–C. All groups exhibited distinct peaks in linear acceleration following impact, with the 8-week group showing the highest peak, followed by the 10-week and 12-week groups (Figure 2A). Linear acceleration peaked at approximately 1.6 milliseconds in all groups before rapidly declining. A similar pattern was observed for rotational acceleration (Figure 2B), where the 8-week group again exhibited the highest peak, followed by the 10-week and 12-week groups. Peak rotational acceleration also occurred around 1.6 milliseconds post-impact. Figure 2C summarizes the peak values of both linear and rotational acceleration, comparing them across groups. An inverse relationship was observed between age and acceleration magnitude. Notably, the 10-week and 12-week groups exhibited significantly lower peak rotational acceleration compared to the 8-week group (*F*(2,21) = 5.1, *p* < 0.05), with the 12-week group also showing significantly reduced peak linear acceleration (*F*(2,21) = 5.7, *p* < 0.05).

**Figure 2:**
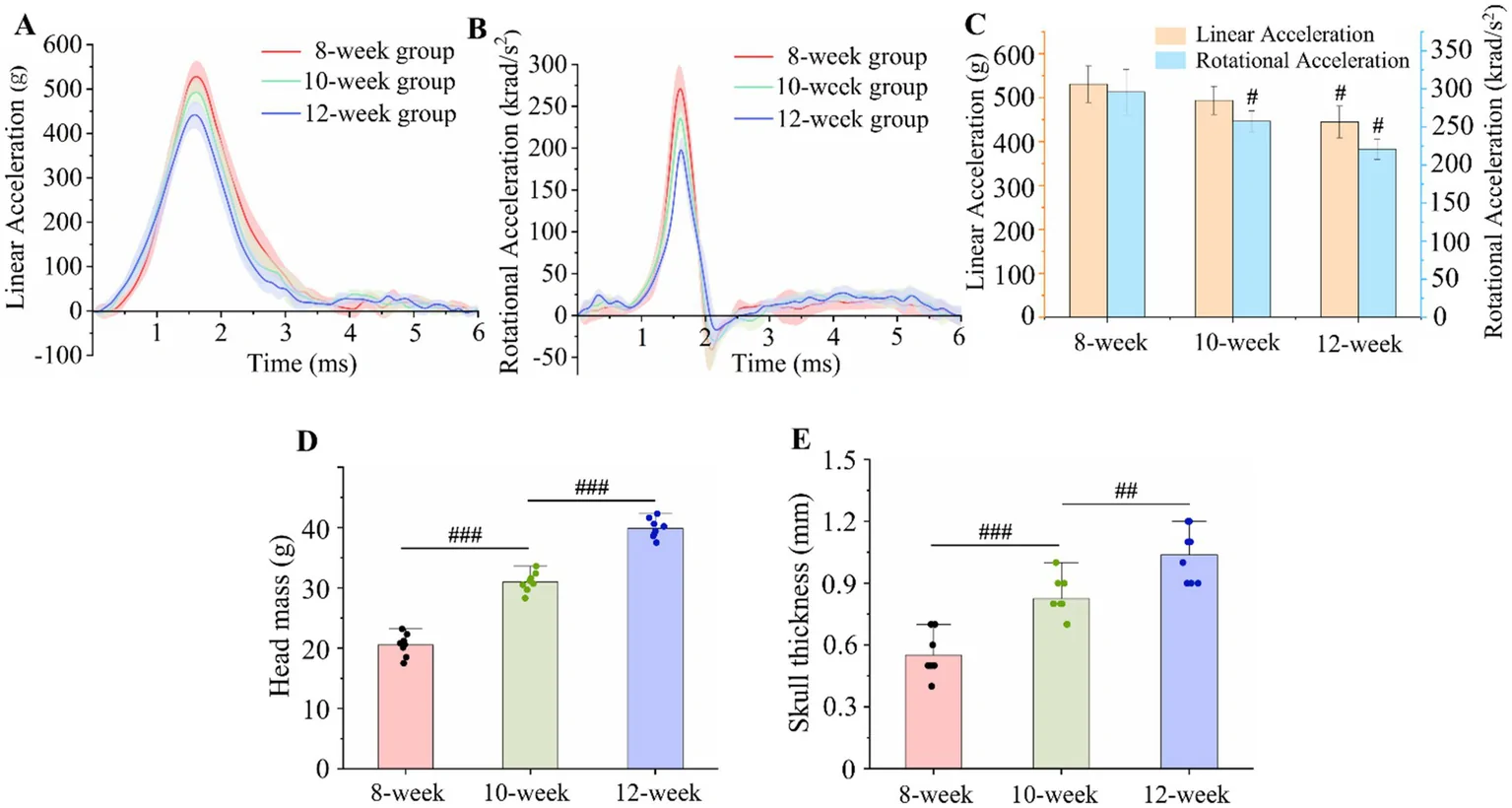
Comparison of head kinematic responses, head mass, and skull thickness among rats of different ages. **(A,B)** Time-history curves of linear and rotational accelerations, with peak values decreasing with age. **(C)** Peak rotational acceleration was significantly lower in the 10- and 12-week groups than in the 8-week group ([*F*(2,21) = 5.10, *p* < 0.05]), whereas peak linear acceleration was significantly lower only in the 12-week group ([*F*(2,21) = 5.70, *p* < 0.05]). **(D)** Head mass differed significantly among age groups ([*F*(2,21) = 16.80, *p* < 0.001]), with higher values in the 12-week group than in the 8- and 10-week groups. **(E)** Skull thickness also differed significantly among groups ([*F*(2,21) = 12.60, *p* < 0.01]), with higher values in the 10- and 12-week groups than in the 8-week group. Data are presented as the mean ± SD in **(C–E)**, whereas the curves in **(A,B)** show the mean with 95% confidence intervals.

The differences in kinematic responses may be related to the head characteristics of the rats. Therefore, further analysis was conducted on the head mass (measured by severing the head at the foramen magnum and then weighing it) and skull thickness (at the impact location), as shown in Figures 2D,E. With increasing age, both head mass and skull thickness significantly increased, with the 12-week-old rats showing notably higher values in both measures compared to the 8-week-old and 10-week-old groups (*F*(2,21) = 16.8, *p* < 0.001, *F*(2,21) = 12.6, *p* < 0.01). Specifically, the 8-week-old rats had the lowest head mass and skull thickness, with significant differences compared to the 10-week-old and 12-week-old groups.

The results demonstrate that under identical impact strength, younger rats exhibited higher peak head accelerations. They further indicate that head mass and skull thickness increased significantly with age and corresponded to the changes in kinematic responses, highlighting that head structural characteristics are important determinants of impact-induced biomechanical responses.

### 3.3 Behavioral changes

Behavioral outcomes are summarized in Figure 3. LRR duration was significantly prolonged in all TBI groups compared with their age-matched sham groups (8 weeks: (*F*(1,14) = 408.86, *p* < 0.001); 10 weeks: (*F*(1,14) = 175.66, *p* < 0.001); 12 weeks: (*F*(1,14) = 132.07, *p* < 0.001); Figure 3A). The 8-week TBI group also showed a significantly longer LRR duration than the 12-week TBI group ([*F*(1,14) = 12.20, *p* < 0.01]), indicating greater impairment of consciousness in the 8-week group.

**Figure 3:**
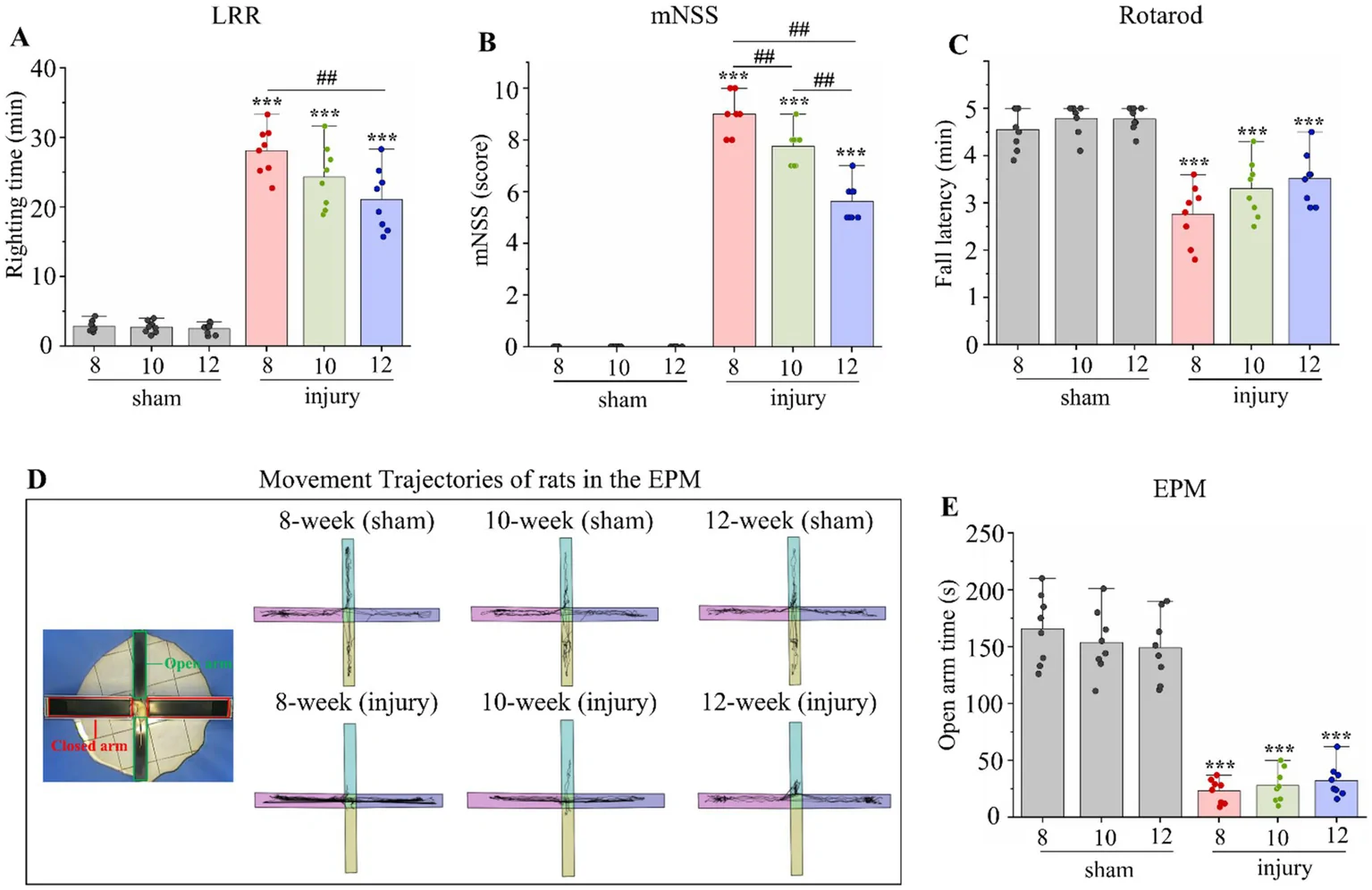
Behavioral outcomes following brain injury in rats of different ages. **(A)** LRR duration increased in all TBI groups versus age-matched sham groups ([*F*(1,14) = 408.86, 175.66, and132.07, respectively; all *p* < 0.001]), and was longer in the 8-week than the 12-week TBI group ([*F*(1,14) = 12.20, *p* < 0.01]). **(B)** mNSS differed among the six groups ([H(5) = 45.69, *p* < 0.001]), with higher scores after injury and lower scores in the older TBI groups. **(C)** Rotarod performance decreased after injury ([*F*(1,14) = 44.30, 37.22, and 34.55; all *p* < 0.001]), without significant differences among TBI groups ([*F*(2,21) = 3.36, *p* = 0.054]). **(D)** Representative EPM trajectories. **(E)** Open-arm time decreased after injury ([*F*(1,14) = 154.47, 126.57, and99.55; all *p* < 0.001]), without significant differences among TBI groups ([*F*(2,21) = 0.94, *p* = 0.407]).

The mNSS differed significantly among the six groups ([*H*(5) = 45.69, *p* < 0.001]). All TBI groups had higher scores than their age-matched sham groups, whereas the 10- and 12-week TBI groups had lower scores than the 8-week TBI group [all (*p* < 0.01); Figure 3B], indicating less severe neurological impairment in the older groups.

Rotarod performance was significantly reduced in all TBI groups relative to their age-matched sham groups (8 weeks: (*F*(1,14) = 44.30, *p* < 0.001); 10 weeks: (*F*(1,14) = 37.22, *p* < 0.001); 12 weeks: (*F*(1,14) = 34.55, *p* < 0.001); Figure 3C), with no significant difference among the three TBI groups ([*F*(2,21) = 3.36, *p* = 0.054]).

In the elevated plus maze, injured rats predominantly remained in the closed arms and spent significantly less time in the open arms than their age-matched sham groups (8 weeks: (*F*(1,14) = 154.47, *p* < 0.001); 10 weeks: (*F*(1,14) = 126.57, *p* < 0.001); 12 weeks: (*F*(1,14) = 99.55, *p* < 0.001); Figures 3D,E), indicating increased anxiety-like behavior. No significant difference was detected among the three TBI groups ([*F*(2,21) = 0.94, *p* = 0.407]).

### 3.4 Histopathology changes

Figures 4, 5 illustrate histopathological changes in the cerebral cortex, hippocampus, and corpus callosum of rats with different ages following brain injury. Figures 4A–D show Nissl staining results, where sham-operated rats displayed intact cortical and hippocampal structures, with neurons exhibiting normal morphology, including plump cell bodies, evenly stained nuclei, and orderly arrangement. In contrast, injured rats demonstrated varying degrees of neuronal pathology, such as shrunken cell bodies, chromatin condensation, blurred cell contours, and vacuolar degeneration (indicated by red arrows), suggesting neuronal degeneration and apoptosis.

**Figure 4:**
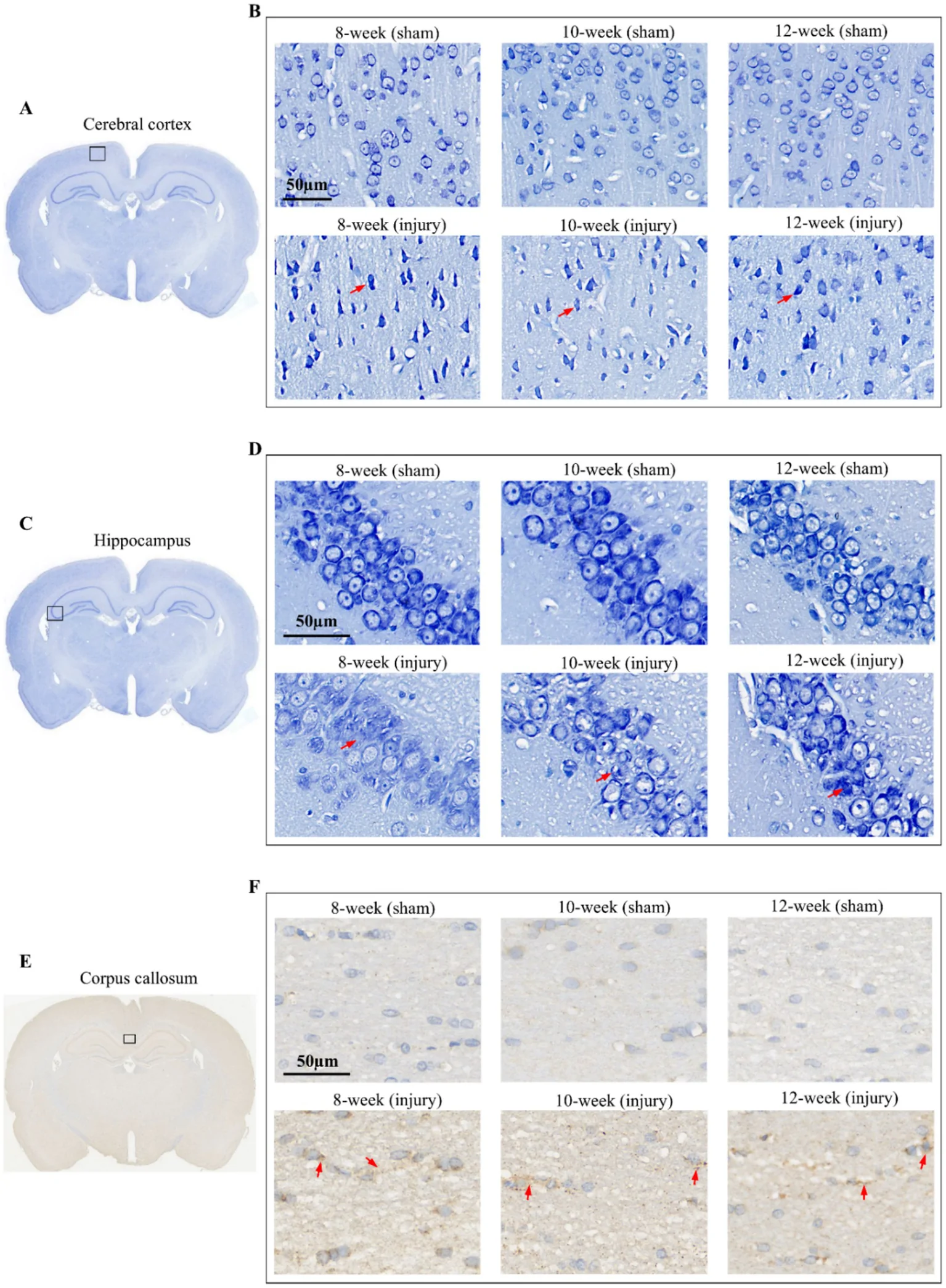
Histopathological changes in brain regions following injury in rats of different ages. **(A–D)** Nissl staining of the cerebral cortex and hippocampus. Injured groups showed neuronal loss, disorganized structure, and degenerative changes, with the most severe damage observed in the 8-week group (red arrows). **(E,F)** β-APP immunostaining in the corpus callosum revealed axonal injury, with the strongest β-APP accumulation in the 8-week group and milder expression in the 10-week and 12-week groups (red arrows).

**Figure 5:**
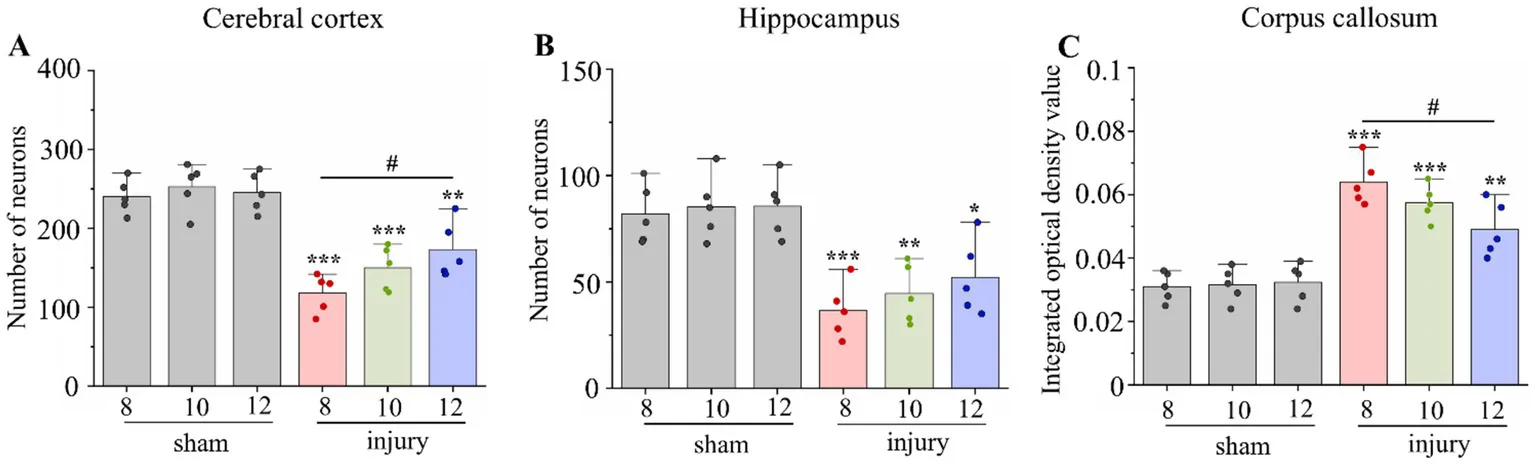
Quantification of neuronal loss and axonal injury in the cerebral cortex, hippocampus, and corpus callosum. **(A)** Cortical neuronal counts decreased after injury ([*F*(1,8) = 71.77, 31.65, and13.79; *p* < 0.001, *p* < 0.001, and *p* < 0.01]), with higher counts in the 12-week than the 8-week TBI group ([*F*(2,12) = 4.39, *p* = 0.037]). **(B)** Hippocampal neuronal counts also decreased after injury ([*F*(1,8) = 27.98, 19.58, and 10.84; *p* < 0.001, *p* < 0.01, and *p* < 0.05]). **(C)** Corpus callosum β-APP IOD increased after injury ([*F*(1,8) = 74.08, 54.92, and 12.27; *p* < 0.001, *p* < 0.001, and *p* < 0.01]), with a lower value in the 12-week than the 8-week TBI group ([*F*(2,12) = 5.39, *p* = 0.021]).

In the cerebral cortex, the 8-week group showed the most severe neuronal loss and disorganization, with some areas nearly devoid of intact neurons. The 10-week group also exhibited evident cell loss and irregular arrangement. The 12-week group showed relatively preserved neuronal morphology, with milder structural disruption. Similar trends were observed in the hippocampal CA3 region: the 8-week group displayed the most severe disorganization and cellular shrinkage, followed by the 10-week group, while the 12-week group retained largely intact neuronal morphology with only mild degeneration in limited areas.

β-APP immunohistochemistry of the corpus callosum (Figures 4E,F) was used to assess axonal injury. Sham groups showed minimal β-APP expression, whereas all injured groups displayed various degrees of β-APP–positive granular deposition (marked by red arrows), seen as brown punctate signals along axonal tracts. The 8-week group exhibited the strongest β-APP immunoreactivity, with dense and widespread deposits; the 10-week group showed moderate expression; and the 12-week group displayed the lowest levels, with sparse deposits, suggesting milder axonal injury.

Quantitative results are presented in Figure 5. Compared with their age-matched sham groups, neuronal counts in both the cerebral cortex and hippocampus were significantly reduced in all injury groups (cortex: 8 weeks, (*F*(1,8) = 71.77, *p* < 0.001); 10 weeks, (*F*(1,8) = 31.65, *p* < 0.001); 12 weeks, (*F*(1,8) = 13.79, *p* < 0.01); hippocampus: 8 weeks, (*F*(1,8) = 27.98, *p* < 0.001); 10 weeks, (*F*(1,8) = 19.58, *p* < 0.01); 12 weeks, (*F*(1,8) = 10.84, *p* < 0.05); Figures 5A,B). Cortical neuronal counts differed significantly among the three injury groups ([*F*(2,12) = 4.39, *p* = 0.037]), with higher counts in the 12-week group than in the 8-week group (Tukey-adjusted (*p* < 0.05)). In the corpus callosum, β-APP integrated optical density was significantly elevated in all injury groups relative to their corresponding sham groups (8 weeks, (*F*(1,8) = 74.08, *p* < 0.001); 10 weeks, (*F*(1,8) = 54.92, *p* < 0.001); 12 weeks, (*F*(1,8) = 12.27, *p* < 0.01); Figure 5C). A significant difference was also detected among the three injury groups ([*F*(2,12) = 5.39, *p* = 0.021]), with a lower value in the 12-week group than in the 8-week group (Tukey-adjusted (*p* < 0.05)); the 10-week group showed an intermediate level.

### 3.5 WB

To characterize molecular changes associated with neuroinflammation, apoptosis, and axonal pathology, GFAP, cleaved caspase-3, and IL-1β expression was examined in the cerebral cortex, hippocampus, and corpus callosum, respectively (Figures 6A–C). Compared with their age-matched sham groups, all three markers were significantly elevated in the 8-week groups (GFAP: (*F*(1,4) = 27.09, *p* < 0.01); cleaved caspase-3: (*F*(1,4) = 27.70, *p* < 0.01); IL-1β: (*F*(1,4) = 22.11, *p* < 0.01)) and 10-week groups (GFAP: (*F*(1,4) = 14.65, *p* < 0.05); cleaved caspase-3: (*F*(1,4) = 27.43, *p* < 0.01); IL-1β: (*F*(1,4) = 11.37, *p* < 0.05)), but not in the 12-week groups (GFAP: (*F*(1,4) = 1.06, *p* = 0.361); cleaved caspase-3: (*F*(1,4) = 3.56, *p* = 0.132); IL-1β: (*F*(1,4) = 0.94, *p* = 0.387)). Significant differences were also detected among the three TBI groups for GFAP ((*F*(2,6) = 6.80, *p* = 0.029)), cleaved caspase-3 ((*F*(2,6) = 7.42, *p* = 0.024)), and IL-1β ((*F*(2,6) = 6.73, *p* = 0.029)); each marker was significantly lower in the 12-week group than in the 8-week group (all Tukey-adjusted (*p* < 0.05)). These findings indicate more pronounced astrocytic activation, apoptotic signaling, and axonal pathology in the younger TBI groups under the tested injury condition.

**Figure 6:**
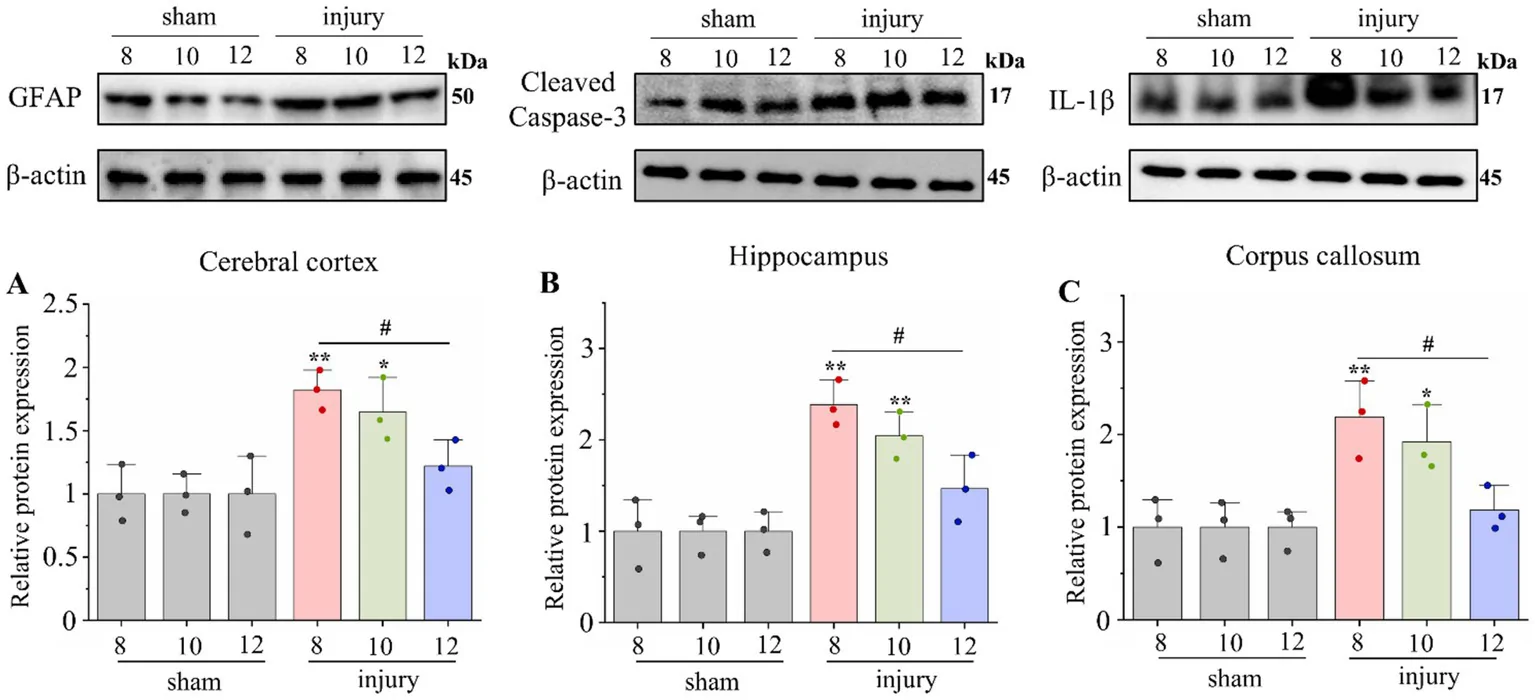
Expression of GFAP, cleaved caspase-3, and IL-β following brain injury in rats of different ages. **(A)** Cortical GFAP increased in the 8- and 10-week TBI groups but not in the 12-week group ([*F*(1,4) = 27.09,14.65, and 1.06; *p* < 0.01, *p* < 0.05, and *p* = 0.361]), with a lower level in the 12-week than the 8-week TBI group ([*F*(2,6) = 6.80, *p* = 0.029]). Similar patterns were observed for **(B)** hippocampal cleaved caspase-3 ([*F*(1,4) = 27.70, 27.43, and 3.56; *p* < 0.01, *p* < 0.01, and *p* = 0.132; *F*(2,6) = 7.42, *p* = 0.024]) and **(C)** corpus callosum IL-β ([*F*(1,4) = 22.11, 11.37, and 0.94; *p* < 0.01, *p* < 0.05, and *p* = 0.387; *F*(2,6) = 6.73, *p* = 0.029]).

## 4 Discussion

TBI is characterized by marked complexity and heterogeneity, and establishing stable and reliable animal models is essential for elucidating its injury mechanisms. The CHI model has been widely used because it can effectively simulate the blunt head trauma commonly seen in clinical settings; however, its outcomes remain susceptible to inter-animal variability. In the present study, we compared injury differences among 8-, 10-, and 12-week-old rats under the same impact intensity and found that 8-week-old rats exhibited the most severe injury, while injury severity gradually decreased with increasing age. These findings indicate that age is an important factor influencing CHI model outcomes and should be strictly controlled in TBI animal experiments.

Our study revealed clear age-related differences in biomechanical and neuropathological responses. Under the same nominal impact setting, the 8-week-old rats exhibited higher linear and rotational accelerations, which may be partly attributable to their lower head mass. Skull thickness may represent an additional structural factor influencing injury transmission (7). In a closed-head impact, the skull is not completely rigid but undergoes transient local deformation, through which the contact load is transmitted to the underlying brain. A thinner skull generally has lower structural stiffness and may therefore undergo greater local flexure under comparable loading, increasing the compressive and strain responses of the brain tissue beneath the impact site. Conversely, the thicker skulls observed in the older rats may have reduced local skull deformation and attenuated this direct loading component. Previous experimental and finite element studies have indicated that skull deformation can increase strain in the cortex and corpus callosum beneath the impact site, and that variations in skull properties can influence head acceleration and regional brain strain (21). Thus, the thinner skulls of the 8-week-old rats may have contributed, together with their lower head mass and greater head kinematics, to the more severe behavioral and neuropathological outcomes observed in this group.

In addition to these biomechanical factors, age-related differences in developmental maturity may also contribute to the observed injury outcomes. Previous studies have shown that the rat brain continues to mature from adolescence to early adulthood, particularly through ongoing neural circuit remodeling and white matter development (24). Accordingly, the more severe injury observed in the 8-week-old rats may also reflect the greater vulnerability of their less mature nervous system, whereas the more developmentally advanced nervous systems of the 10- and 12-week-old rats may have greater tolerance to traumatic insult. These findings suggest that age reflects not only differences in physical characteristics but also changes in neurobiological maturity.

The present findings are also consistent with evidence from other animal models. In a mouse closed-head injury model, adolescent mice exhibited greater neurodegeneration and behavioral deficits than adult mice under the same injury condition, indicating greater vulnerability of the developing brain (18). Similarly, Zvejniece et al. found that older mice had thicker parietal bones and a lower incidence of skull fracture following weight-drop injury, whereas skull fracture was associated with greater neurological impairment and inflammatory responses (17). Together, these findings support age as an important source of variability in rodent TBI models. However, the relationship between age and clinical TBI outcomes is more complex and is influenced by developmental stage, injury severity, comorbidities, and recovery time. Therefore, the present findings should not be interpreted as indicating that increasing age is universally protective against TBI in humans; rather, age-related differences should be considered when interpreting and translating experimental TBI findings.

These region-specific assessments of the cortex, hippocampus, and corpus callosum were not intended to provide redundant descriptions of the same injury, but rather to characterize the sequential pathological processes following TBI from different perspectives. Morphological changes in neurons mainly reflect the structural endpoint of cellular injury in gray matter, whereas GFAP and cleaved caspase-3 correspond to astrocytic reactivity and activation of apoptotic pathways after injury, respectively. Therefore, the histopathological damage observed in the cortex and hippocampus is biologically consistent with the changes in cortical GFAP and cleaved caspase-3. Previous studies have shown that reactive astrocytes emerge rapidly after TBI and are accompanied by increased GFAP expression (25); meanwhile, caspase-3 activation contributes to neuronal death in the cortex and hippocampus (24), which is consistent with the interpretation of the present findings.

As a major white matter tract, the corpus callosum is particularly vulnerable to axonal injury following TBI and was therefore selected to evaluate white matter pathology (26). β-APP accumulation reflects impaired axonal transport and provides histopathological evidence of axonal injury (27). In addition, IL-1β is a key pro-inflammatory cytokine that is upregulated after TBI and may contribute to secondary white matter injury by amplifying the local inflammatory response (28). Thus, the combined assessment of β-APP and IL-1β in the corpus callosum enabled evaluation of both structural axonal damage and the associated neuroinflammatory response. The greater β-APP accumulation and IL-1β expression observed in the younger rats suggest that their more pronounced axonal injury was accompanied by a stronger inflammatory response, which may partly contribute to the age-related differences in injury outcomes.

Although this study provides valuable insights into the impact of different adolescent ages on TBI outcomes, there are several limitations. First, a single nominal impact setting of 0.7 MPa was used to isolate age-related differences in head kinematics and injury outcomes under a standardized input. However, this design did not allow assessment of the interaction between age and injury intensity or determine whether the observed pattern applies to milder injuries. Future studies incorporating multiple impact intensities are needed to establish age-specific intensity-response relationships. Second, the molecular assessment was limited to GFAP, cleaved caspase-3, and IL-1β, which reflected age-related differences in astrocyte activation, neuronal apoptosis, and axonal pathology but could not identify the underlying regulatory pathways. Future transcriptomic and proteomic analyses, combined with targeted validation, are needed to clarify the roles of microRNAs, cytokines, and regeneration-related factors in age-dependent TBI responses. Third, the present study focused on acute-phase responses and did not include longitudinal assessments of neurological function, brain structure, or neuropathological changes. Therefore, the current findings cannot determine whether the observed age-related differences persist, diminish, or change during recovery. Future studies incorporating multiple follow-up time points are needed to compare the trajectories of functional recovery, neuronal survival, and axonal pathology among rats of different ages. Fourth, no analgesic medication was administered after injury, and pain was not independently quantified using a pain-specific assessment scale. Although the procedure did not involve craniotomy or skin incision, post-impact pain or stress cannot be excluded and may have affected behavioral performance and pathological responses. Future studies should incorporate validated pain-assessment methods and evaluate analgesic regimens that minimize interference with neurological, behavioral, and molecular outcome. Lastly, the present study did not assess systemic metabolic disturbances, oxidative stress, or peripheral inflammatory responses. Consequently, the current results cannot determine whether age-related differences in energy metabolism, redox homeostasis, or peripheral immune activation contributed to the observed differences in acute TBI outcomes. Future studies integrating brain tissue analysis with measurements of metabolic, oxidative stress, and circulating inflammatory markers are needed to provide a more comprehensive characterization of age-dependent responses to TBI.

## 5 Conclusion

In conclusion, this study demonstrates the significant impact of adolescent age on the severity of TBI in rats, with younger animals (8 weeks of age) exhibiting more severe neurological impairments, neuronal loss, and axonal injury compared to older rats (10 and 12 weeks of age). The findings suggest that age-related differences in brain biomechanics, neuroinflammatory responses, and tissue repair mechanisms contribute to these variations in injury severity. These results underscore the importance of considering age as a critical factor in preclinical TBI research and highlight the need for age-specific models to improve the translational relevance of therapeutic interventions.

## Data Availability

The raw data supporting the conclusions of this article will be made available by the authors, without undue reservation.
